# Transcriptome-based variations effectively untangling the intraspecific relationships and selection signals in Xinyang Maojian tea population

**DOI:** 10.3389/fpls.2023.1114284

**Published:** 2023-02-20

**Authors:** Lin Cheng, Mengge Li, Yachao Wang, Qunwei Han, Yanlin Hao, Zhen Qiao, Wei Zhang, Lin Qiu, Andong Gong, Zhihan Zhang, Tao Li, Shanshan Luo, Linshuang Tang, Daliang Liu, Hao Yin, Song Lu, Tiago Santana Balbuena, Yiyong Zhao

**Affiliations:** ^1^ Henan International Joint Laboratory of Tea-oil tree Biology and High Value Utilization, Xinyang Normal University, Xinyang, Henan, China; ^2^ College of Life Sciences, Xinyang Normal University, Xinyang, Henan, China; ^3^ Laboratory of Systematic Evolution and Biogeography of Woody Plants, School of Ecology and Nature Conservation, Beijing Forestry University, Beijing, China; ^4^ Institute of Forestry Science, Xinyang Forestry Bureau, Xinyang, Henan, China; ^5^ College of Engineering and Technology, Northeast Forestry University, Harbin, China; ^6^ College of Agriculture, Guizhou University, Guiyang, China; ^7^ Department of Agricultural, Livestock and Environmental Biotechnology, Sao Paulo State University, Jaboticabal, Brazil

**Keywords:** *Camellia sinensis*, tea, transcriptome, SNPs, phylogeny, population genetics, nucleotide diversity, selective sweep

## Abstract

As one of the world’s top three popular non-alcoholic beverages, tea is economically and culturally valuable. Xinyang Maojian, this elegant green tea, is one of the top ten famous tea in China and has gained prominence for thousands of years. However, the cultivation history of Xinyang Maojian tea population and selection signals of differentiation from the other major variety *Camellia sinensis* var. *assamica* (*CSA*) remain unclear. We newly generated 94 *Camellia sinensis* (*C*. *sinensis*) transcriptomes including 59 samples in the Xinyang area and 35 samples collected from 13 other major tea planting provinces in China. Comparing the very low resolution of phylogeny inferred from 1785 low-copy nuclear genes with 94 *C. sinensis* samples, we successfully resolved the phylogeny of *C. sinensis* samples by 99,115 high-quality SNPs from the coding region. The sources of tea planted in the Xinyang area were extensive and complex. Specifically, Shihe District and Gushi County were the two earliest tea planting areas in Xinyang, reflecting a long history of tea planting. Furthermore, we identified numerous selection sweeps during the differentiation of *CSA* and *CSS* and these positive selection genes are involved in many aspects such as regulation of secondary metabolites synthesis, amino acid metabolism, photosynthesis, etc. Numerous specific selective sweeps of modern cultivars were annotated with functions in various different aspects, indicating the *CSS* and *CSA* populations possibly underwent independent specific domestication processes. Our study indicated that transcriptome-based SNP-calling is an efficient and cost-effective method in untangling intraspecific phylogenetic relationships. This study provides a significant understanding of the cultivation history of the famous Chinese tea Xinyang Maojian and unravels the genetic basis of physiological and ecological differences between the two major tea subspecies.

## Summary

The source of tea plant populations cultivated in Xinyang are elusive even though Xinyang Maojian is one of the ‘top ten famous tea’ in China. We presented 94 newly sequenced transcriptomes of *Camellia sinensis*, and 99,115 high-quality SNPs were identified. We successfully untangled the intraspecific relationships of tea plants in central China based on transcriptomic variations. We found that the sources of tea planted in Xinyang were extensive and complex. Shihe District and Gushi County were the two earliest tea planting areas. Furthermore, we identified numerous putative selective-sweep genes during the differentiation between two subspecies of *Camellia sinensis* involved in the regulation of secondary metabolites synthesis.

## 1 Introduction

The tea plant (*Camellia sinensis* (L.) O. Kuntze, 2n = 2x = 30) is a member of the Theaceae family, angiosperm order Ericales. It is one of the significant economic woody crops worldwide, covering a cultivated area of more than 4.08 million hectares across more than 60 tea-cultivated countries, especially in South China, East Asia, Africa, and Latin America (http://www.fao.org) (Wang et al., 2020). The tender shoots and leaves of the tea plant can be used to produce tea, the second most popular non-alcoholic beverage, following water (Rietveld and Wiseman, 2003). Tea boasts important health benefits owing to its accumulated abundant secondary metabolites, including theanine, catechin, and caffeine (Wan and Xia, 2015). The unique tea plant variety is the prerequisite to high-quality tea produced by different processing technology according to the processing suitability (PS) (Zhang et al., 2020a). In addition to its unique flavors, tea has been verified to have some compounds with positive bioactive effects on human health (Zhao et al., 2021a). *Camellia sinensis* var. *sinensis* (*CSS*) and *Camellia sinensis* var. *assamica* (*CSA*) are the two main tea plant cultivated groups worldwide. *CSS* is mainly distributed in colder and warm climate zones with small leaves and lower shrubs. *CSA* has large leaves growing rapidly, and is mainly planted in tropical and subtropical areas (Wei et al., 2018). Different biological characteristics endow *CSS* and *CSA* with significant differences in a wide variety of secondary metabolites that contribute to the tea quality, including flavor, taste, fragrance, and tea color, making them have different suitability (Zeng et al., 2019).

**Figure 1:**
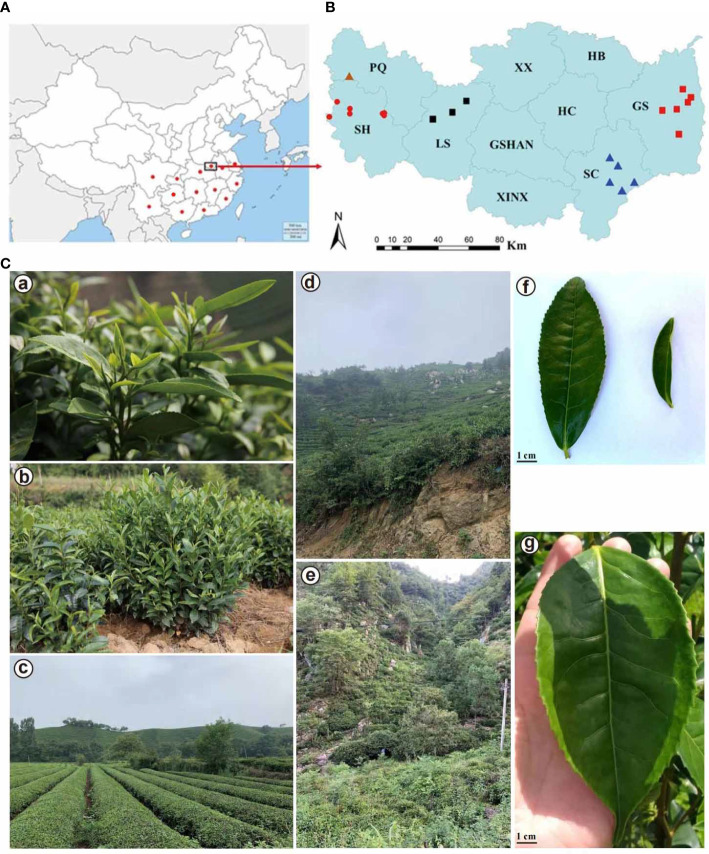
Geographical distribution of 94 *Camellia* accessions, leaf morphology and habitat of the Xinyang Maojian tea population. **(A)** Geographical distribution of 94 *Camellia* samples. Red dots indicate *Camellia* samples in different provinces of China. **(B)** Geographical distribution of 59 *Camellia sinensis* var. *sinensis* from central China, Xinyang city, Henan province. PQ, Pingqiao District; SH, Shihe District; LS, Luoshan County; XX, Xi County; GSHAN, Guangshan County; XINX, Xin County; HB, Huaibin County; HC, Huangchuan County; GS, Gushi County; SC, Shangcheng County. Dots with the same shapes indicate the same sampling location. **(C)** Leaf morphology and habitat of the Xinyang Maojian tea populations. **(a)** Leaf morphology of the Xinyang Maojian tea population. **(b)** The close-up view of the tree or shrub life-form of Xinyang Maojian tea population. **(c, d)** indicate the habitat of Xinyang Maojian tea population. **(e)** Habitat of the Xinyang Maojian wild tea population with more than 200 years of cultivation history. **(f)** Different tea leaf morphologies in Xinyang city. **(g)** Leaf morphology of *Camellia sinensis* var. *assamica*.

Xinyang is located in the south of Henan Province (32°N, 114°E) (**Figures 1A, B**), between the upper-middle Huaihe River, Tongbai Mountain, and Dabie Mountain. The terrain is high in the south and low in the north, with a ladder-like banding distribution of mountainous areas in the southwest, hilly areas in the middle, and plain depressions in the north successively. It is also a transitional climate zone from subtropical to temperate regions. The main tea-producing areas in Shihe and Pingqiao District of Xinyang including Cheyun Mountain (CY), Jiyun Mountain (JY), Yunwu Mountain (YW), Tianyun Mountain (TY), Lianyun Mountain, Heilongtan (HLT), Bailongtan (BLT), and Hejiazhai (HJZ). The other main tea-cultivating counties in Xinyang including Gushi county (GS), Shangcheng county (sc), and Luoshan county (LS). Xinyang Maojian is one of the ‘top ten famous tea’ in China, with its long history dating back to the Zhou Dynasty (BC 1046-256) and flourishing in the Tang (AD 618-907) and Song Dynasty (AD 960-1279) (Yu, AD780). Xinyang Maojian tea is famous for its particularity of ‘slender, round, tight, straight, multi-trichome, high fragrance, strong flavor, and green color’. Compared with other noted green tea, it is rich in polyphenols, catechins, amino acids, and caffeine and also contains abundant microelements (Cui et al., 2022b). The different contents and proportions of biochemical components in tea plant leaves contribute to the unique quality flavor and determine the PS to a great extent (Jia, 2013). As one of the remarkable green teas, Xinyang Maojian tea shows its own PS according to previous studies (Cui et al., 2022a). The leaves from ‘Xinyang No. 10’ from the Xinyang District and ‘Fuding Dabai’ from Fujian Province could endow the tea soup with a high amino acid content, moderate tea polyphenols, and high caffeine content, and represent the optimum quality with light green color and fresh taste (Jia, 2013). In contrast, ‘Wuniuzao’ and ‘Shuchazao’ contained fewer tea polyphenols, amino acids, caffeine, and more phenol-ammonia, and produced yellow-green tea soup with a bland fragrance, leading to poorer quality green tea products (Jia, 2013). However, the sources of tea plants grown in this area still need to be explored due to its long cultivation history and complicated donor regions of introduction, making it challenging to select and develop improved varieties. Therefore, it is of great significance to figure out the source of varieties and populations of cultivated tea plants, which will lay the theoretical foundations for the subsequent reconstruction of poor-quality tea gardens and upgrading of tea quality.

Plant traits have been modified by humankind due to their benefits, including large fruit or grain size (Shomura et al., 2008), lower plant height, and reduced seed shattering (Li et al., 2006; Ishikawa et al., 2022), which made cultivated groups distinguish from its wild relatives (Li et al., 2019). Annuals such as rice (*Oryza sativa*) (Huang and Han, 2015; Yu et al., 2021), maize (*Zea mays*) (Stitzer and Ross-Ibarra, 2018; Chen et al., 2021), common bean (*Phaseolus vulgaris*) (Gaut, 2014), potato (*Solanum tuberosum*) (Kyriakidou et al., 2020), and tomato (*Solanum lycopersicum*) (Sato et al., 2012) were domesticated initially, while long-lived perennial including trees were domesticated afterwards (Gunn et al., 2011; Miller and Gross, 2011). The common bean exhibited a significant decrease in nucleotide diversity of coding sequence (60%) and gene expression (18%) compared with wild progenitors (Gaut, 2014). The same trend was also evident in rice and soybean (Lam et al., 2010; Xu et al., 2012). In addition, 7% of the maize genome was shown to have undergone artificial selection during the history of domestication (Hufford et al., 2012). While for long-lived perennials, the domestication of apple and peach have been studied through resequencing at the whole genome levels (Duan et al., 2017; Wu et al., 2018), some candidate genes associated with domestication traits including fruit size and sugar content have been detected (Wu et al., 2018). As an ancient tree crop, the tea plant has a long cultivation history of nearly thousands of years (Zhang et al., 2021). During this period, the interaction between tea plants and environments, the farmer selection, all driven tea plant domestication for better flavor (Zhang et al., 2021). Some domestication traits as well as their related genes have been identified (Xia et al., 2020b). In addition, *CSS* and *CSA* had been evidenced of parallel domestication, and some genes were artificially selected in the early domestication processes (Zhang et al., 2021). However, due to the long history of cultivation and a large number of tea plant landraces in China, it is of great significance to further explore domestication-related genes associated with characteristic flavor formation of *CSS* and *CSA* by adding more landraces data.

With the development of high-throughput sequencing technology, especially the second-generation sequencing technology represented by Illumina, the cost of sequencing has significantly decreased, and phylogenetic relationships were reconstructed using more complete data (Grabherr et al., 2011). One of the first long sequences put into use was chloroplast genomes. Because of its extremely large number of copies in cells, it can be obtained with low sequencing depth (Sun et al., 2018; Lloyd-Evans et al., 2019). However, this approach still exposes some flaws: (1) the phylogenetic analyses by chloroplast gene fail to detect hybridization events due to its uniparental inheritance; (2) chloroplast genes are very conservative and the informative loci in chloroplasts are rare in some radiating evolution groups (Davis et al., 2014). Only by using sequences with more informative sites, such as nuclear genes, can the phylogenetic relationships of reticulate groups be better solved (Huang et al., 2016a). At present, the following methods have been extensively employed to obtain numerous nuclear genome data, including exon capture (Ng et al., 2009), reduced-representation genome sequencing (RRGS) (Baird et al., 2008), genome resequencing (Bentley, 2006), genome-skimming (Liu et al., 2022), and transcriptome sequencing (Zhang et al., 2022). However, no universally recognized optimal technology has been developed, and each technology has its advantages and defects, applicable to different biological levels (Johnson et al., 2019). When a study focuses on lower levels such as subgenera, the above technical methods can be used in principle. However, the experimental cost and data output need to be taken into consideration. The lower price of transcriptome sequencing makes it more ideal than other techniques (Zeng et al., 2017). In addition, orthologous genes (OGs) can be obtained from the transcriptome dataset and are suitable for systematic analyses at different classification levels (Zhao et al., 2021b). Single nucleotide polymorphism (SNP) mainly refers to DNA sequence polymorphism induced by single nucleotide variation at the genome level (Leaché et al., 2015). This molecular marker has been widely used in crop genetic analysis owing to its high density, genetic stability, and easy automation (Kobayashi et al., 2020; Zhang et al., 2020b). The whole-genome resequencing was performed for further SNP calling on ancient tea plant samples (Wang et al., 2020; Yu et al., 2020; Zhang et al., 2020d). However, the SNP-calling based on the transcriptome data for intraspecific relationship analyses in tea plants has not been reported.

The objectives of this study were to construct a transcriptomic variations method for intraspecies relationship analysis, and to investigate the selective sweep-related genes in tea plants. We presented 94 newly sequenced transcriptomes of *Camellia sinensis* in different regions of Xinyang and other main tea-producing provinces in China. Comparing the very low resolution of phylogeny inferred from 1785 low-copy nuclear genes with 94 *C. sinensis* samples, we successfully resolved the phylogeny of *C. sinensis* samples by 99,115 high-quality SNPs from the coding region. We found that the cultivation of tea plants in the Xinyang area is extensive and complex, implying the long history of tea planting in this area. Our study offered an effective approach for untangling intraspecific relationships based on the transcriptomic SNP-calling approach in *Camellia sinensis*, and classification was carried out on the unknown tea plants belonging to *CSS* or *CSA*. Functional investigations of the selective sweep-related genes in tea plants have been applied. This study will provide a significant theoretical basis for its processing suitability and lay a foundation for the excavation of valuable wild germplasm resources in tea plants.

## 2 Materials and methods

### 2.1 Taxon sampling, sequencing, and transcriptome assembly

A total of 94 young leaves of *Camellia* were collected from 14 provinces, including Anhui, Chongqing, Fujian, Guangdong, Guangxi, Guizhou, Hubei, Hunan, Jiangsu, Jiangxi, Sichuan, Zhejiang, Yunnan, and Henan of China (**Figure 1A**). The newly collected young leaves were frozen in liquid nitrogen and quickly stored in an ultra-low temperature refrigerator (-80 °C). In terms of transcriptome sequencing, the total RNA was extracted from young tissues using a modified TRIzol method (Hummon et al., 2007). Transcriptomes were sequenced using the Illumina BGISEQ-500 platform with paired 100 bp.

Low-quality reads were filtered out by SOAPnuke v1.5.2 (Chen et al., 2018) (https://github.com/BGI-flexlab/SOAPnuke). All transcriptomes were *de novo* assembled into contigs using Trinity v2.11.0 (Grabherr et al., 2011). Moreover, TransDecoder v5.5.0 (http://transdecoder.sourceforge.net/, accessed June 2022) was used for CDS region prediction, and redundant contigs from each assembly were reduced using CD-HIT 4.8.1 with the parameter of -c 0.98 as described in the previous studies (Fu et al., 2012; Huang et al., 2016a; Xiang et al., 2017; Zeng et al., 2017; Qi et al., 2018). The software BUSCO v5.2.2 was utilized to evaluate the gene completeness for each annotation using the eudicots_odb10 database (Simão et al., 2015). The information on transcriptomes generated in this study and BUSCO assessment of assembly completeness were described in **Supplementary Table S1**.

### 2.2 Orthologs identification

The reservoir of 1785 putative low-copy nuclear genes used in this study was identified from the previous study (Cheng et al., 2022b). Generally, OGs were identified with OrthoFinder v2.0.0 (Emms and Kelly, 2019) through 11 genomes of 8 families. The 11 genomes include *Lactuca sativa* (Reyes-Chin-Wo et al., 2017), *Chrysanthemum seticuspe* (Hirakawa et al., 2019), *Daucus carota* (Iorizzo et al., 2016), *Solanum lycopersicum* (Hosmani et al., 2019), *Capsicum annuum* (Kim et al., 2014), *CSS* ‘Shuchazao’ (Wei et al., 2018), *CSA* ‘Yunkang 10’ (Xia et al., 2017), *Actinidia chinensis* (Wu et al., 2019), *Primula veris* (Nowak et al., 2015), *Vitis vinifera* (Jaillon et al., 2007), and *Aquilegia coerulea* (Filiault et al., 2018). The resulting 1785 OGs were used as source genes to obtain the corresponding putative orthologs (E-value < 1e-20) from 94 new assemblies of transcriptomes in HaMStR v13.2.6 (Ebersberger et al., 2009). The numbers of low-copy nuclear genes identified by HaMStR from those 1785 OGs were described in **Supplementary Table S1**.

### 2.3 Transcriptome-based variant calling

To further investigate the reliability of transcriptome data for phylogeny inference, the variant calling was carried out following the best practice workflow for RNA-seq short variant discovery in Genome Analysis Toolkit (GATK) (McKenna et al., 2010). In addition to the 94 newly sequenced transcriptomes, the other 14 public RNA-seq data were also included and detailed information for these public transcriptomes is in **Supplementary Table S2**. The trimmed reads from each transcriptome were mapped to the reference genome using BWA-MEM v.0.7.17 with default parameters (Li and Durbin, 2009; Xia et al., 2020b). The high-quality chromosome-level reference genome is a diploid elite cultivar of *CSS* ‘Shuchazao’ (2n = 2x = 30 chromosomes) (Xia et al., 2020b). The aligned bam files were sorted and indexed using SAMtools v.1.12 (Li et al., 2009). The ‘MarkDuplicates’ in GATK v.4.2.5 was used to mark the potential PCR duplicated reads (McKenna et al., 2010). Subsequently, the ‘HaplotypeCaller’ was employed to output intermediate GVCFs of each individual, and ‘CombineGVCFs’ was adopted to merge all the GVCF files into a single GVCF. The raw variants were generated using ‘GenotypeGVCFs’ implemented in GATK. The hard filtering was performed to filter SNPs using ‘VariantFiltration’ and ‘SelectVariants’ of GATK with the parameter of “QD < 10.0 || FS > 60.0 || MQ < 40.0 || SOR > 3.0 || MQRankSum < -12.5 | and | ReadPosRankSum < -8.0”. To further remove false positive SNPs, genotype calls with a depth lower than one-third or higher than two-fold of the average depth (DP) were removed. Finally, only biallelic variants with a missing data rate of less than 10% and a minor allele frequency (MAF) over 0.02 were retained. The SNP density was plotted using R packages “CMplot” (Yin et al., 2021).

### 2.4 Phylogeny inference and principal component analysis

With the development of high-throughput sequencing technology, more and more nuclear genes of species are obtained for phylogenomic analyses. Phylogenetics inference through large-scale genes concatenated into a supermatrix has proven to be flawed, such as being prone to systematic errors (and artifacts) and leading to an inaccurate phylogenetic relationship (Philippe et al., 2017). To understand the evolutionary history of tea plant populations cultivated in Xinyang, we constructed their phylogeny from both coalescent and ML methods by using low-copy nuclear genes and SNPs, respectively.

For coalescent analyses with 1785 low-copy nuclear genes, 94 new assemblies of sampled *C. sinensis* and two genome data (*CSA* ‘Yunkang 10’ and *CSS* ‘Shuchazao’) (Xia et al., 2017; Xia et al., 2020b) were used for phylogeny construction. Amino acid sequences were aligned using MAFFT v7.487 (Katoh and Standley, 2013) with the “-auto” parameter. Poorly aligned regions were further trimmed using the trimAl v1.2 (Capella-Gutiérrez et al., 2009) with the “-automated1” parameter. Multiple amino acid sequence alignments were converted to nucleotide alignments by PAL2NAL (Suyama et al., 2006). Single-gene ML trees were reconstructed using IQ-TREE v2.1.4-beta (Nguyen et al., 2015) under the GTR+ G model with 1000 bootstrap replicates. The coalescent analysis was implemented by ASTRAL.5.7.8 (Zhang et al., 2018).

For ML analyses by concatenating SNPs, a total of 108 samples included the 94 newly sequenced transcriptomes, and the RNA-seq data of *CSA* ‘Yunkang 10,’ and *CSS* ‘Biyun,’ ‘Hangdan,’ ‘Tieguanyin,’ ‘Longjing43’ and ‘Shuchazao,’ (Xia et al., 2017; Wang et al., 2020; Xia et al., 2020b; Zhang et al., 2020c; Wang et al., 2021b) and eight wild tea species (**Supplementary Table S2**). The SNP dataset was converted to a PHYLIP file using the Python script ‘vcf2phylip’ (https://github.com/edgardomortiz/vcf2phylip/, accessed June 2022). The ML phylogeny was also inferred using IQ-TREE (Nguyen et al., 2015). The optimum model was selected with the maximum Bayesian Information Criterion (BIC) scores estimated by ModelFinder (Kalyaanamoorthy et al., 2017) implemented in IQ-TREE. Principal component analysis (PCA) was performed using Plink v1.90b6.25 (Purcell et al., 2007).

### 2.5 Analyses of nucleotide diversity and genome-wide scan for selection signatures in coding region

The 99,115 high-quality SNPs constructed a high-quality genetic map of the coding region of *Camellia* germplasm resources. To identify genomic regions that may contain variants selected during the differentiation of *CSA* and *CSS* populations, we identified regions that were highly diverged between three *CSA* samples and 97 *CSS* accessions by testing selection signals from per loci (VCFtools-based method) and genomic blocks (XP-CLR based method). The genes detected by both methods were considered reliable and robust selection signals for further functional annotation analyses.

Nucleotide diversity (π) was estimated from 20-kb windows across the genome for *CSA* and *CSS* populations by VCFtools (Danecek et al., 2011). This study examined positive selection by scanning both single locus and genomic blocks to obtain comprehensive and reliable genes with selection signatures. VCFtools (Danecek et al., 2011) was used to calculate population Fst statistic as previously described between different populations (Cagan and Blass, 2016; Lee et al., 2020; Wang et al., 2021a) and the per-site Fst value was calculated between two populations with the parameter “–weir-fst-pop”. The R packages “GeneNet” (Schaefer et al., 2015) was implemented to get the Z-transform Fst value for visualization in Manhattan plots by the R package “CMplot” in **Figure 5**. The genes with the top 10% of Fst value were considered selective sweeps. These top-ranked genes were used for further GO (Ashburner et al., 2000) and KEGG (Kanehisa and Goto, 2000) annotation and enrichment analyses.

Cross-population composite likelihood ratio test was performed by comparing the genetic diversity of SNPs loci in the above coding regions between *CSS* and *CSA* using XP-CLR software (Chen et al., 2010). Genomic regions of significant inclusion selective sweep were identified using a sliding window of 20-kb scanning of the genome. Selection sweeps can increase genetic differentiation between populations, causing allele frequencies to deviate from the expected values under neutral conditions. XP-CLR modeled the multi-locus allele frequency differentiation between the two populations, and used Brownian motion to simulate genetic drift under neutral conditions. Approximately, deterministic models were used to perform selective scanning for nearby single nucleotide polymorphisms (SNPs). *CSA* and *CSS*-specific genomic blocks under selection signals were calculated by XP-CLR (Chen et al., 2010) for selective sweeps using the parameters of “–ld 0.95 –phased –maxsnps 600 –size 200000 –step 20000”. The top 1% positive XP-CLR values of *CSS* and *CSA*-specific genomic blocks under selection were identified as selective sweeps. The genes located in these genomic blocks under selection were used for further GO and KEGG annotation and enrichment analyses.

### 2.6 Gene ontology and KEGG annotation and enrichment analyses

To speculate the gene functions, we referred to the annotation results of *CSS ‘Shuchazao’* in 2020 by Xia et al. (2020b). The analyses of GO and KEGG were performed using the OmicShare tools, a free online platform for data analysis (https://www.omicshare.com/tools, accessed June 2022). The gene function annotation of GO and KEGG of the chromosome-level genome of *CSS* were retrieved from Tea Plant Information Archive (TPIA, http://tpdbtmp.shengxin.ren:81/, accessed June 2022) as the background file for enrichment analyses.

### 2.7 Gene family analyses and detection of gene duplication events

To investigate whether gene duplications (GDs) contribute to the diversification between *CSS* and *CSA* populations, 53 selected genes with selection signals identified by both VCFtools and XP-CLR in 11 representative genomes (*CSA ‘Yunkang 10’*; *CSS ‘Biyun’, ‘Hangdan’, ‘Tieguanyin’, ‘Longjing43’* and *‘Shuchazao’*; *Camellia lanceoleosa*, *Camellia oleifera* var. *‘Nanyongensis’*, *Camellia* DASZ*, Actinidia chinensis, Rhododendron simsii*) were conducted for gene family analysis. The 51 of 53 genes mentioned above were used for GD analysis except for *CSS0023764* and *CSS0046895* (the total number of homologs < 4).

The homologous proteins were identified by BlastP with E-value 1e-5 from the above-mentioned representative genomes. Subsequently, amino acid sequences were aligned using MAFFT v7.487 (Katoh and Standley, 2013) with the “-auto” parameter. Poorly aligned regions were further trimmed using the trimAl v1.2 (Capella-Gutiérrez et al., 2009) software with the “-automated1” parameter. Multiple amino acid sequence alignments were converted to nucleotide alignments by PAL2NAL (Suyama et al., 2006) software. The ML gene family trees were constructed using IQ-TREE v2.1.4-beta (Nguyen et al., 2015) under the GTR+G model with 1000 bootstrap replicates. The gene duplication events were detected by tree2gd (https://github.com/Dee-chen/Tree2gd/, accessed on 20 May 2022) with the parameters “–bp=70 –sub_bp=70 –species=2”. Finally, The R packages “pheatmap” was implemented to create the heat maps showing the numbers of homologs and GD events across the phylogeny proposed in our previous study (Kolde, 2019; Cheng et al., 2022b).

## 3 Results

### 3.1 Distribution of *Camellia* samples collected in Xinyang

RNA sequencing was conducted on 94 tea accessions collected from 14 provinces of China (**Figure 1A**). Among these tea accessions, 33 were registered cultivars, and 61 were of uncertain identity. A total of 59 tea accessions were collected from five different administrative areas, including Shihe District, Pingqiao District, Luoshan County, Shangcheng County, and Gushi County (**Figure 1B**). The tea trees cultivated in the Xinyang District are generally *CSS*, a lower-growing shrub with a small leaf (**Figures 1C, a–f**), capable of withstanding colder climate compared to *CSA* (Assam type) (**Figure 1C, g**). The majority of areas in Xinyang are yellow-brown loam with fertile soil (**Figures 1C, b, d**). Additionally, the other natural conditions, including light, temperature, and water, are suitable for *CSS* growth as well. The total area of tea gardens in these five districts accounted for 80% of the tea garden total area in Xinyang, representing diverse cultural environments and altitudes. For example, Shihe District and Pingqiao District had almost plain or hills with low mountains with an average altitude of 150-300 m (**Figures 1C, b, c**). The tea trees cultivated in Shangcheng County and Gushi County were almost distributed in high mountains with an average altitude of 550 m (**Figures 1C, d, e**).

### 3.2 Generation of 94 new tea accession transcriptome datasets and SNP-calling

**Figure 2:**
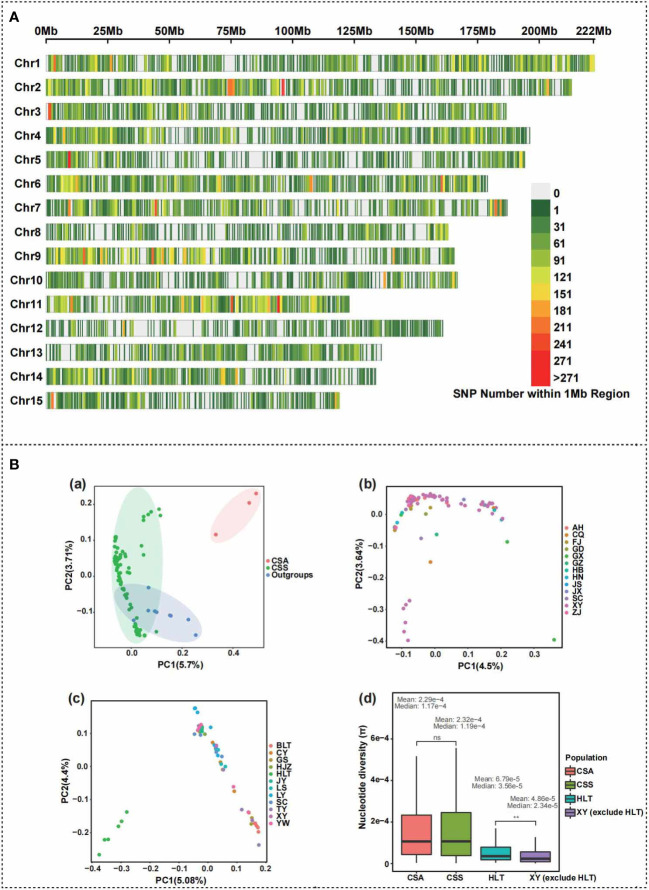
Principal component analyses and genetic diversity calculation by SNPs of 108 *Camellia* samples. **(A)** Distribution and density map of identified SNPs on 15 chromosomes of *Camellia sinensis* var. *sinensis*. **(B)** Principal component analysis (PCA) of 108 *Camellia* accessions for samples clustering. **(a)** The result of PCA of 108 *Camellia* samples. CSS indicate *Camellia sinensis* var. *sinensis* population; CSA indicates *Camellia sinensis* var. *assamica* population. Red, green and blue ellipses represent CSA, CSS and wild tea population, respectively. **(b)** The result of PCA of 92 *Camellia sinensis* var. *sinensis* samples. AH, Anhui; CQ, Chongqing; FJ, Fujian; GD, Guangdong; GX, Guangxi; GZ, Guizhou; HB, Hubei; HN, Hunan; JS, Jiangsu; JX, Jiangxi; SC, Sichuan; XY, Xinyang; ZJ, Zhejiang. **(c)** The result of PCA of 59 *Camellia sinensis* var. *sinensis* samples from Xinyang City. BLT, Bailongtan; CY, Mount Cheyun; GS, Gushi County; HJZ, Hejiazhai; HLT, Heilongtan; JY, Mount Jiyun; LS, Luoshan County; LY, Mount Lianyun; SC, Shangcheng County; TY, Mount Tianyun; XY, Xinyang; YW, Mount Yunwu. **(d)** The comparison of nucleotide diversity between *Camellia sinensis* var. *sinensis* (CSS) with *Camellia sinensis* var. *assamica* (CSA) population, and between Xinyang Heilongtan (CSS HLT) with Xinyang except for Heilongtan (XY except for HLT) samples. The mean and median nucleotide diversity values were above the box for each tea population. The results of the significance tests are indicated using ns (no significant difference) and ** (p < 0.01).

The number of 94 individual filtrated reads ranged from 37,192,224 to 46,890,150, the total number of bases shifted from 5,578,833,600 to 7,033,522,500, and the mapping rate of *CSS* reference genomes changed from 93.02% to 99.05% (**Supplementary Table S1**). The transcriptomic data of 94 samples were newly measured using Trinity, TransDecoder, and CD-HIT for *de novo* assembly, new transcript prediction, and quality assessment using BUSCO in our study. The number of new transcripts ranged from 14,648 to 20,516; the average length changed from 1172.33 to 1256.59 bp; Contig N50 varied from 1527 to 1629 bp; BUSCO (C) shifted from 57.5% to 67.1% (**Supplementary Table S1**). The data quality was good for further phylogenomic and population genomic analyses. After the strict filtration, a total of 99,115 high-quality SNPs were obtained for further analyses. The flowchart for transcriptome-based SNP calling, phylogeny analysis and selection signals identification is described in **Supplementary Figure 1**. These SNPs were not evenly distributed among the chromosomes (**Figure 2A**). Chromosome1 consisted of the majority of 9063 SNPs, while chromosom11 had more SNPs with one variant per 17,199 bp. The SNP density of 12 distinct regions greater than 200/Mb was identified and dispersive at nine chromosomes. On average, there are about 35 variants per Mb. Principal component analysis indicated that the *CSS* accessions from Xinyang City, Henan Province, were grouped with other *CSS* accessions, and the *CSA* accessions were separated from *CSS* individuals (**Figure 2B, a**). In addition, the accessions from HLT were separated from all the other individuals of *CSS* (**Figures 2B, b, c**). The nucleotide diversity of the population of HLT was significantly higher than other populations collected in Xinyang (**Figure 2B, d**). The results of the nucleotide diversity analysis suggest that the tea at HLT may be a relatively original core tea germplasm resource in Xinyang. The higher the genetic diversity, the more genes are available in the community for environmental selection, and the more adaptable the community is to the environment, which is conducive to the survival and evolution of the community. In summary, we were able to obtain sufficient SNPs based on transcriptome data to support our exploration of population evolutionary history at the intraspecies level.

### 3.3 Low-copy nuclear genes by the coalescent method failed to resolve the intraspecies relationships of tea plant cultivars

**Figure 3:**
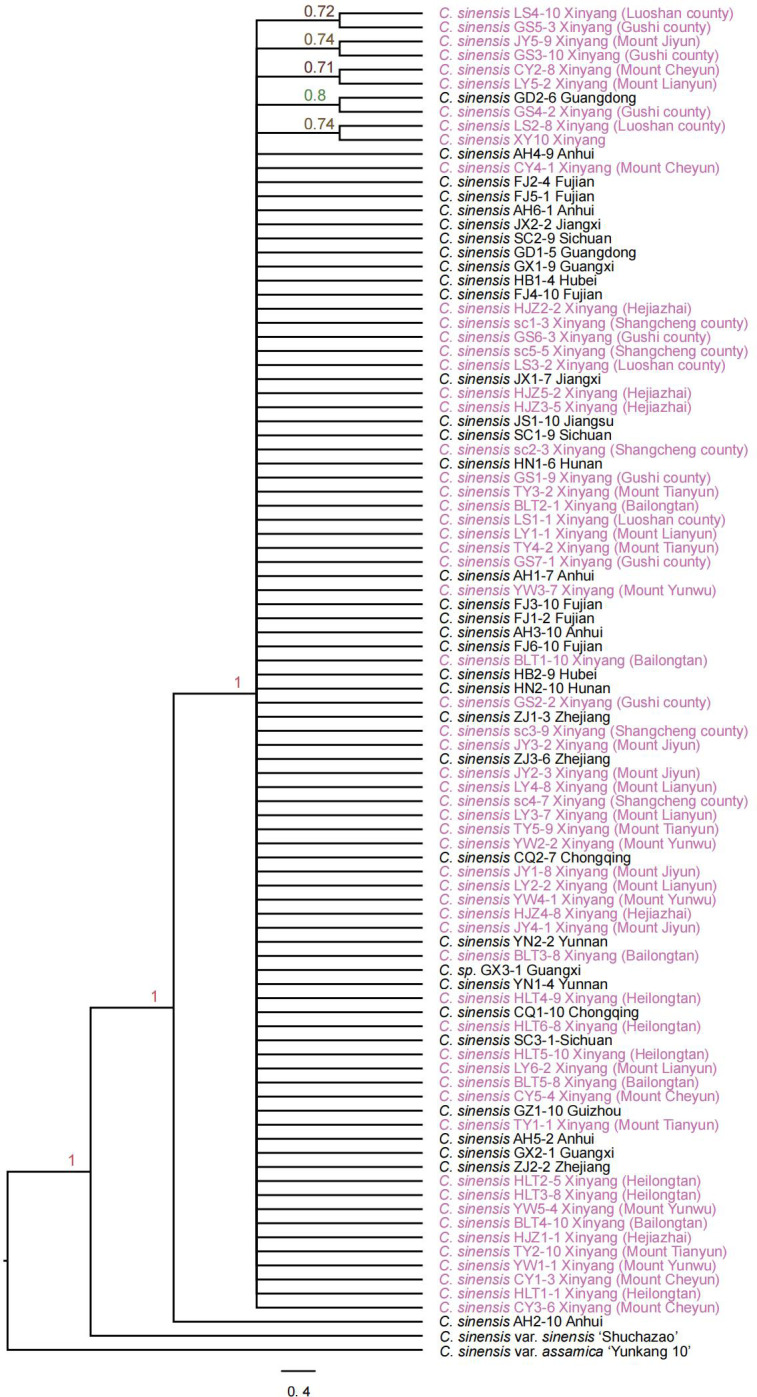
The coalescent phylogenetic tree was inferred from 94 *Camellia* samples with 1785 low-copy nuclear genes. The nodes with posteriori probability (PP) less than 0.7 were collapsed into a comb. The species names with purple color indicate samples collected from Xinyang City.

A total of 1785 low-copy nuclear orthologous genes were identified from 94 *Camellia sinensis* samples generated in this study as well as two public genomes *C. sinensis* var. *sinensis* ‘Shuchazao’ and *C. sinensis* var. *assamica* ‘Yunkang 10’ as outgroup. The IQ-TREE v2.1.4-beta package was used to reconstruct the ML gene family tree with the GTR+G model and 1000 bootstrap replicates. The alignments of 428 OGs were highly conservative and showed few informative sites for inference of phylogenetic relationships. The coalescent phylogenetic trees were inferred from 1357 low-copy nuclear genes with 96 *C. sinensis* samples and a node with less than 0.7 local posterior probability was collapsed into a comb (**Figure 3**). Finally, we get a species tree with poor support, with 89% of the nodes having support values below 0.7 from coalescent analysis (**Figure 3**). The poorly resolved phylogeny with numerous comb structures indicates that the coalescent method with low-copy nuclear genes could not resolve the intraspecies relationships in *Camellia sinensis*. Low-copy nuclear genes are somewhat conserved and do not have sufficient informative sites among subspecies populations, resulting in the inability to resolve their phylogenetic relationships by the coalescent method.

### 3.4 Transcriptomic variation effectively untangled intraspecific relationships in *Camellia sinensis*

**Figure 4:**
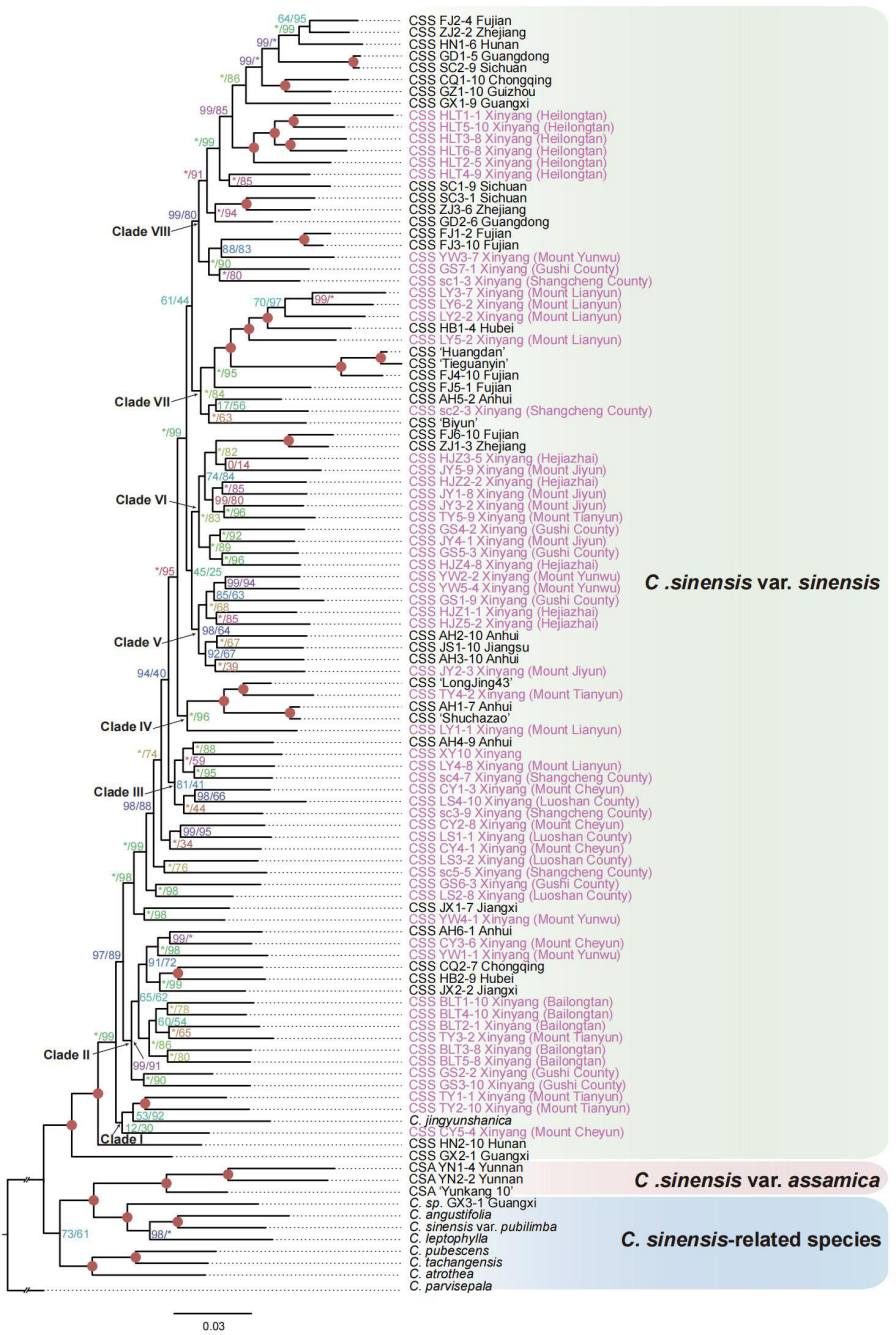
The maximum likelihood (ML) phylogenetic tree was inferred from a concatenated supermatrix of 99115 SNPs. The ML tree was reconstructed using IQ-TREE with best-fit model TVM+F+R4 and ultrafast bootstrap (UFBoot) and H-aLRT test each with 1000 bootstrap replicates. The red circle indicates that the support value of the UFBoot and H-aLRT tests are both 100. The support values of H-aLRT (left) and UFBoot (right) were separated by slashes in the nodes. The asterisk indicates that either H-aLRT (left) or UFBoot (right) support value is 100. CSS, *Camellia sinensis* var. *sinensis*; CSA, *Camellia sinensis* var. *assamica.* The purple color was used to mark the samples collected in the Xinyang area.

The coalescent method failed to resolve the relationships within the 94 *Camellia sinensis* populations from 1785 low-copy nuclear genes. Our results indicate that low-copy nuclear genes are too conserved and do not contain sufficient phylogenetic informative sites at the intraspecific level. Therefore, we proposed the transcriptome-based SNP callings method to reconstruct the phylogenetic relationship of *C. sinensis* intraspecies based on the ML method. We obtained a highly supported phylogeny of *C. sinensis* populations based on the SNP data and 95% of nodes have bootstrap support values greater than 92 (**Figure 4**). Our phylogeny maximumly supports the representatives of *CSA* subspecies samples as a monophyletic group. Our study strongly supports *CSS* accessions clustered together with *C. jingyunshanica* and this topology was also supported by the previous study (Wu et al., 2022; Cheng et al., 2022b). Our research showed that the tea plants in Guangxi and Hunan were differentiated significantly within *CSS* samples. As the first divergent lineage of *CSS* group, Clade I contained three samples collected in the Shihe District (Xinyang area), which were clustered with *Camellia jingyunshanica*. All these accessions in the Clade I were belonged to the family Theaceae, genus *Camellia*, Sec. *Thea*., but *Camellia jingyunshanica* belongs to Ser. Gymnogynae, and the other three accessions, TY1-1, TY2-10, and CY5-4, were collected in Mountain Tianyun and Cheyun in Xinyang, were belonged to the Ser. Sinenses Chang (**Figure 4**). According to previous study, *C. jingyunshanica* of Ser. Gymnogynae was close to species of *C. assamica* than CSS (Wu et al., 2022; Cheng et al., 2022b). We speculated that the three accessions were situated between the *CSS* and *CSA*. In Clade II, Clade V, and Clade VI, the Shihe District and Gushi County populations clustered together and were sister groups with varieties from other provinces (**Figure 4**). Therefore, speculation was proposed that the ShiHe District and Gushi County were the two earliest tea planting areas in Xinyang, with abundant tea plant population resources and more frequent exchange of tea plant germplasms than other districts. Tea plants from Luoshan County, Shihe District, and Shangcheng County were clustered in Clade III, and ‘Xinyang No. 10’ were clustered with tea plants from Anhui Province. TY4-2 from Tianyun Mountain and ‘Longjing 43’ were clustered together in Clade IV, suggesting TY4-2 possibly belongs to the populations of ‘Longjing 43’. Also, in this clade, AH1-7 from Anhui Province was the sister group to the ‘Shuchazao’ genome data, supporting AH1-7 possibly a population of ‘Shuchazao’. The Shihe District populations in Clade VII and VIII were clustered together with Shangcheng County. They then were successively sister groups to the tea plants from Hubei, Anhui, Fujian, and Sichuan Province, respectively. In addition, principal component analyses of *Camellia* samples (**Figure 2B, c**) support a significant differentiation among tea plant populations of Heilongtan (HLT) in the Shihe District, providing rich genetic diversity for tea plant variety improvement.

### 3.5 Numerous selective sweeps were detected from transcriptome-based SNP-calling approach

**Figure 5:**
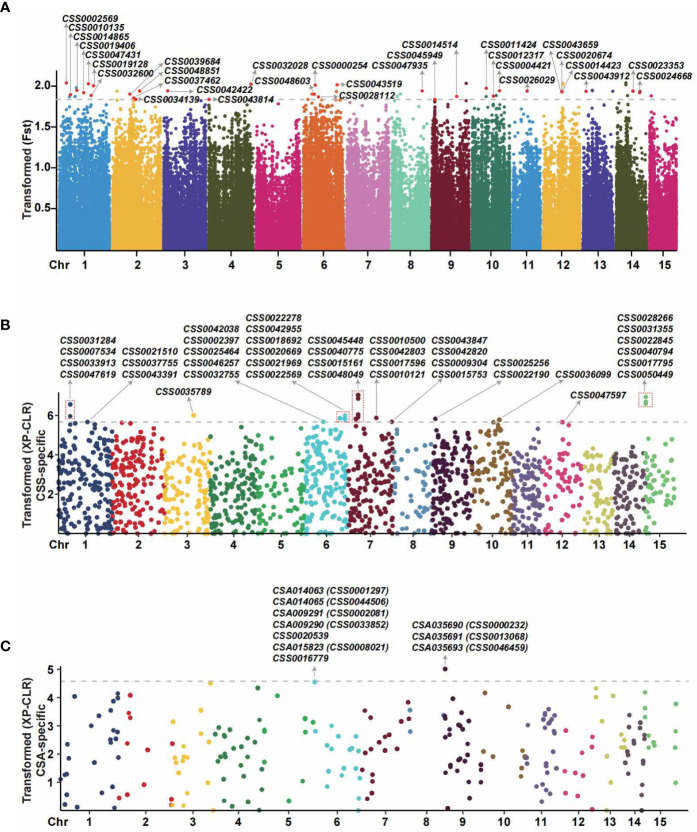
Genome-wide scan for positive selection signatures from modern tea cultivars from the coding region by VCFtools and XP-CLR methods. **(A)** Detection of selection signals based on single loci. The dots of different colors represent SNP sites. The red dots above the grey dashed line represent SNPs with strong positive natural selection signals (top 10% Fst values). Arrows indicate the gene IDs with the detected strong selection signal. The X-axis indicates the chromosome position, and the values of the Y-axis are the transformed fixation index (Fst). **(B)** Detection of the CSS-specific positive natural selection genes from multi-locus (genomic blocks). The dots with different colors represent genomic blocks. The dots above the grey dashed line represent genomic blocks with strong positive natural selection signals (top 1% positive XP-CLR values). The X-axis indicates the chromosome position, and the Y-axis is the log-transformed XP-CLR value. **(C)** The CSA-specific positive natural selection genes were detected from multi-locus (genomic blocks). The dots with different colors represent genomic blocks. The dots above the grey dashed line represent genomic blocks with strong positive natural selection (top 1% positive XP-CLR values). The X-axis indicates the chromosome position, and the Y-axis is the log-transformed XP-CLR value. The BLASTp software was used to identify the orthologs of CSA and CSS with protein sequence identity > 60%.

**Figure 6:**
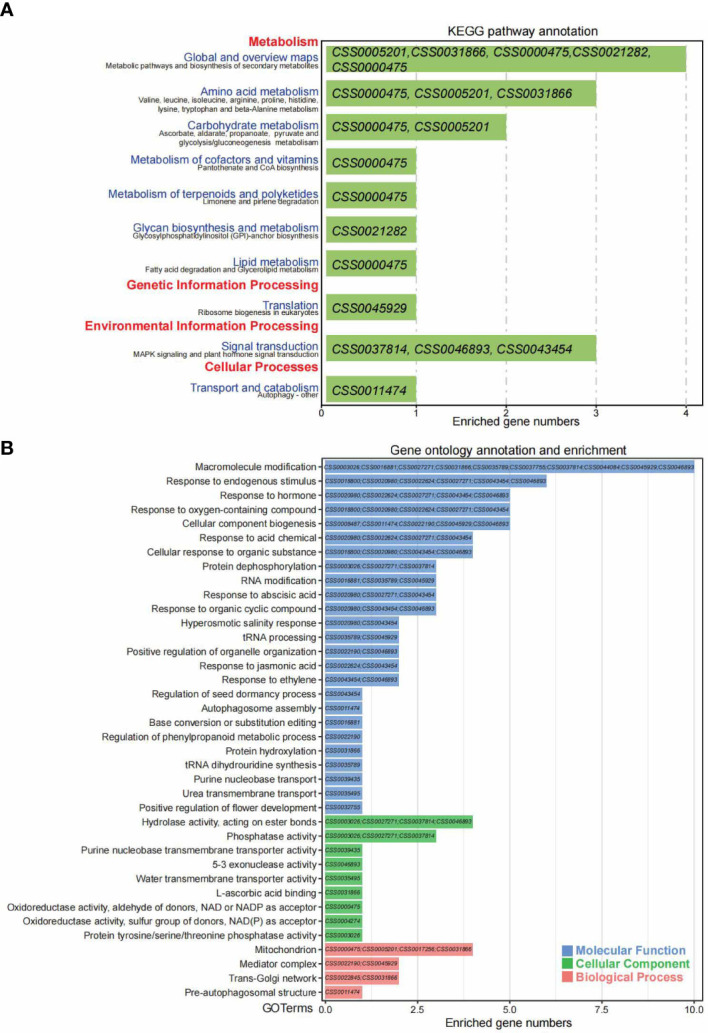
The KEGG and GO annotation and enrichment analyses of overlapped 53 genes under positive natural selection were identified both by single locus and genomic blocks. **(A)** The KEGG pathway annotation and enrichment for 53 overlapped genes with strong selection signals by VCFtools and XP-CLR software. The pathway terms of KEGG_A_class and KEGG_B_class were indicated with red and purple colors on Y-axis, respectively. The gene IDs enriched for each pathway were shown in the green bars. The X-axis indicates enriched gene numbers. **(B)** The GO enrichment analysis for 53 overlapped genes with strong selection signals by VCFtools and XP-CLR software. The GO terms with blue, green and red colors indicate molecular function, cellular component and biological process, respectively. The gene IDs enriched for each GO term were shown in the bars. The X-axis indicates enriched gene numbers.

The program package of VCFtools was used to measure the genetic differentiations between *CSS* with *CSA* at a single variant locus. As shown in **Figure 5A**, a total of 66 SNPs loci with significant population differentiation were obtained, located at 31 genes in total (**Supplementary Table S3**). A number of the genes associated with the domestication of modern tea cultivars have been previously identified, including the genes of *CSS0029726*, *CSS0043088*, *CSS0047361*, *CSS0041258*, *CSS0017987*, *CSS0004406* (Xia et al., 2020b; Li et al., 2022), and notably, we also identified signals for those genes with the Fst value > 0.25. To avoid missing positive selection signals, XP-CLR was also used to detect selective sweeps between populations of *CSS* and *CSA* by genetic differentiation analyses from multiple variant loci (20-kb genomic block). As shown in **Figures 5B, C**, a total of 5680 *CSS*-specific and 1046 *CSA*-specific genomic blocks were detected with selection signal (XP-CLR value > 0) in the XP-CLR likelihood test. The sliding window size of 20 kb of *CSS*-specific and *CSA*-specific genomic blocks have an average of 26.98 and 16.12 SNPs variants, respectively. In detecting selection signals, the more input accessions the population has, the more selective sweeps are likely to be detected. According to the sampling numbers 93 to 3 for the two population groups of *CSS* and *CSA*, we selected the top 1% of *CSS*-specific blocks and *CSA*-specific blocks as the significant blocks. In summary, a total of 86 significant genomic blocks with high XP-CLR values were detected, including 74 *CSS* or 12 *CSA*-specific selective blocks. We detected 41 *CSS*-specific positive selective genes (**Supplementary Table S4**, line F) from 74 significant blocks with XP-CLR values ranging from 49.56 to 131.02 (**Supplementary Table S4**) and 10 *CSA*-specific positive selective genes (**Supplementary Table S5**, line F) from 12 blocks with XP-CLR values ranging from 22.49 to 31.30 (**Supplementary Table S5**). As the two methods for identifying selective sweeps use different algorithms and the population samplings have a large bias, we did not observe an overlap between the 41 and 10 genes described above. To find the positively selected genes detected by both software, we expand the range of significant genes by the two methods to find the robust selective signals. A total of 53 genes overlapped between 509 genes (Fst > 0.9 by VCFtools) and the top 500 genes with high XP-CLR values (407 *CSS*-specific genes with XP-CLR value > 17 and 105 *CSA*-specific genes with XP-CLR value > 9.5). Finally, a total of 53 overlapping genes were obtained from both software, including *CSS0000232, CSS0004274, CSS0005201*, etc. (**Supplementary Table S6**). Additionally, KEGG and GO functional annotations and enrichment analyses of selection signals detected from the software VCFtools, XP-CLR and both were performed, respectively (**Supplementary Figures 2**, **3**, **Figure 6**).

### 3.6 Functional annotation and enrichment of genes under selection signals subjected to the differentiation of the populations of *CSA* and *CSS*

To understand the gene functions, KEGG/GO annotation and enrichment analyses were performed on genes with strong selection signals by VCFtools (31 genes, **Supplementary Figure 2**), XP-CLR (51 genes, **Supplementary Figure 3**) and intersection part (53 genes, **Figure 6**). The KEGG annotation for 31 genes with strong selection signals from VCFtools mainly enriched in the metabolic pathways, arginine biosynthesis, biosynthesis of secondary metabolites, amino acids biosynthesis, fatty acid metabolism, carbon metabolism and photosynthesis, etc. (**Supplementary Figure 2A**). GO enrichment analyses suggested that these genes under selective sweeps significantly enriched in biological process (including nucleoside phosphate biosynthetic process, photosynthesis, ATP synthesis, etc.), molecular function (including ATPase activity, phosphate ion binding, etc.) and cellular component (including chloroplast, photosynthetic membrane, thylakoid, photosystem, etc.) (**Supplementary Figure 2B**). The KEGG annotation for 51 genes with strong selection signals from XP-CLR mainly enriched in the metabolic pathways and plant-pathogen interaction, oxidative phosphorylation and metabolism of glutathione, pyrimidine and purine, etc. (**Supplementary Figure 3A**). GO enrichment analyses suggested that these genes under selective sweeps significantly enriched in biological process (including intracellular transport, proteolysis, cell morphogenesis, mitochondrial-related function, secondary metabolic process, pollen exine formation, etc.), molecular function (including ubiquitin-protein transferase activity, etc.) and cellular component (e.g., mitochondrial respiratory chain complex) (**Supplementary Figure 3B**).

For the shared 53 genes with selection signals detected by both methods, the KEGG enrichment analysis indicates these genes were mainly involved in four major KEGG_A_class terms, metabolism (global and overview maps: biosynthesis of secondary metabolites, amino acid metabolism, carbohydrate metabolism, metabolism of cofactors and vitamins, metabolism of terpenoids and polyketides, glycan biosynthesis and metabolism and lipid metabolism), genetic Information processing (translation: ribosome biogenesis in eukaryotes), environmental information processing (signal transduction: MAPK signaling and plant hormone signal transduction) and cellular processes (transport and catabolism: autophagy). Genes enriched under each term were listed in **Figure 6A**. Besides, gene ontology enrichment analysis revealed that these 53 genes functionally play important roles in 38 terms/pathways, including 25 terms in molecular function, nine in cellular components and four in biological processes. Genes enriched under each term were listed in **Figure 6B**. Briefly, the significantly enriched terms of molecular function including macromolecule modification, response to endogenous stimulus, response to the hormone, response to oxygen-containing compound, and cellular component biogenesis, response to acid chemical, positive regulation of organelle organization, purine nucleobase transport and positive regulation of flower development, etc. The significantly enriched terms in the cellular component include hydrolase activity, phosphatase activity, purine nucleobase transmembrane transporter activity, water transmembrane transporter activity, and protein tyrosine/serine/threonine phosphatase activity, etc. For the biological processes, only four terms were significantly enriched, including mitochondrion, mediator complex, trans-Golgi network and pre-autophagosomal structure (**Figure 6B**).

### 3.7 Genes subject to selective sweeps undergone gene duplication event(s) enhancing different evolutionary directions for *CSA* and *CSS*

To test whether gene duplications (GDs) contributed to the diversification between *CSS* and *CSA* populations, we constructed gene family trees of 51 genes subjected to selective sweeps for gene duplication and gene copy number variation analyses. The 51 genes that overlapped under positive selection identification by both VCFtools and XP-CLR were used for GD analysis except for *CSS0023764* and *CSS0046895* (the total number of homologs < 4). As shown in **Supplementary Figure 4A**, 28 genes have multi-copies across the selected 11 genomes, whereas 23 genes are much more conservative with fewer copies. Several genes with selection signals differed significantly in copy number variation between the *CSA* and *CSS* populations, including genes *CSS0012202*, *CSS0000475*, *CSS0000831, CSS001320* and *CSS0042955*, which may explain the differences between these two populations. The phyto 8 and phyto 9 were the most recent common ancestors (MRCA) of Theaceae and Ericales, respectively (**Supplementary Figure 4**). Numerous genes under selection signals have occurred gene duplication events in the ancestors of Theaceae and Ericales (**Supplementary Figure 4B**). So many genes subject to selective sweeps have undergone ancient gene duplication events at different nodes that may have played important roles in the diversification of tea plants.

In addition, we found that tea plants have recently undergone frequent tandem gene duplication events in different degrees, which might be an important driving force for the enhancement of natural selection or domestication by humankind. Specifically, there is only one copy of *CSS0000831* in the *CSA* species, but three copies in *CSS*_BY. According to the GO annotation, *CSS0000831* was annotated to play important roles in flower development (GO:0048441), primary metabolic process (GO:0044238), and cellular component organization (GO:0016043). The gene of *CSS0013210* was annotated to play an important role in molecular function as acid-amino acid ligase activity (GO:0016881). And The copy numbers of *CSS0013210* vary greatly between the species. In *CSS*, *CSS0013210* expanded by tandem duplication, with ten in *CSS*_TGY, two in *CSS*_BY but only one copy in *CSA*. Some genes also had undergone GDs in *CSS* but without functional annotations, including *CSS0042955*, *CSS0000232*, *CSS0008548* and *CSS0021282*, which required functional investigations (**Supplementary Figure 4C**).

## 4 Discussion

### 4.1 Accurate identification of *CSS* and *CSA* is an important prerequisite for improving tea processing suitability

As an important economic woody crop, tea plants were divided into two main varieties, *CSS* and *CSA* (Wang et al., 2020). *CSS* is characterized by small leaves, shrub or semi-shrub growth habits, and great cold tolerance (Wei et al., 2018), cultivated worldwide owing to their economic values and properties. In contrast, *CSA* is famous for its large leaf size and outstanding tolerance for drought and high temperature (Xia et al., 2017). In addition to their biological differences, *CSS* and *CSA* also have different processing suitability. Generally, green tea is made by *CSS*, while *CSA* is usually provided for making black tea or dark tea (Zeng et al., 2019). However, many environmental factors, including temperature, light, water, and the different growth processes of the plant, will affect the morphologies of plants (Givnish, 1987). The leaf area is one of the key factors affecting the growth and functions of plants and could change dramatically to adapt to environmental changes. For example, the seedling leaf areas of *Dalbergia sissoo* decreased by 67% under drought stresses compared to regular irrigation (Singh and Singh, 2009). Xinyang is located in the transitional zone between subtropic and temperate zones. According to our analyses, all of the 59 samples collected in the Xinyang belonged to *CSS*, even though some populations have relatively large leaf areas (**Figure 1**). Light is another important environmental factor that affects leaf area. According to the tradeoff mechanism, shade plants always have larger leaf areas than plants growing in sunlight. For instance, as an important understory component of the lowland and montane forests in the subtropical regions of Asia and South America, dwarf bamboo leaves in the shaded area were thinner than those in the open area (Yang et al., 2014). We predicted that some of the *CSS* populations in Xinyang have large leaf area mainly because they were planted in shaded areas. The long-term lack of lights induced a greater leaf area than before. On the contrary, the samples YN1 and YN2 were *CSA* populations from Yunnan Province, even though both YN1 and YN2 have small leaf areas, similar to the classic *CSS*. Therefore, leaf morphology, especially the leaf area, cannot be the only criterion for species classification. Here, we proposed an effective approach to untangle the intraspecific relationship based on transcriptome SNP-calling and we can accurately classify the unknown tea plants as *CSS* or *CSA*. Precise classification of the subspecies type of tea provides an essential theoretical basis for its processing suitability.

### 4.2 Accurate species and core population identification is essential to unlock the genetics potential of local tea germplasm resources

Cultivating tea plant varieties with special characters could promote the additional value of tea products. The development and utilization of elite germplasm resources is also a hot spot in the tea industry. Xinyang is the main tea-producing area in the north of the Yangtze River in China, with a long history of tea production and rich germplasm resources of tea plants. Meanwhile, Xinyang is in the subtropics and warm temperate zone, which is the transitional zone between the north and south climates, with a great position in the introduction, adaptation and domestication of tea plants. The germplasm resources of the local tea plant in Xinyang are formed under long-term natural and artificial selection, which have strong adaptability to the local environment and numerous resistance and tea quality-related genes evolved. According to our analysis, the continuous introduction of seeds or elites of the tea plant from other provinces and asexual propagation of cuttings has led to tea plant cultivation in the Xinyang becoming more confusing (**Figure 4**). In the meantime, with the promotion of superior clones, local populations are facing the danger of being eliminated. Narrowed genetic diversity among modern cultivars may greatly restrict the subsequent creation of elite cultivars (Zhou et al., 2015; Liu et al., 2020). In our study, the nucleotide diversity of HLT populations was significantly higher than the other samples in Xinyang. Protection and utilization of populations with genetic diversity is essential for both functional studies and breeding (Huang et al., 2015). At present, the source of existing germplasm resources is unknown, and some germplasm resources have been lost, which is extremely unfavorable to the cultivation of tea plant breeding. In order to understand the populations of tea plants in Xinyang more clearly, a more in-depth and comprehensive study is required, and considering the high level of genetic diversity present in the local populations needs to be sequenced in the future.

### 4.3 Transcriptome-based SNP-calling is an efficient method to solve intraspecific relationships in *Camellia sinensis*

The phylogenies based on low-copy nuclear genes were widely accepted to reconstruct more robust phylogenetic relationships at different taxonomy levels, including order, family, and genus levels (Zeng et al., 2014; Huang et al., 2016b; Zhao et al., 2021b; Cheng et al., 2022a). Based on our analysis, low-copy nuclear genes cannot provide efficient information for the reconstruction of intraspecies relationships in tea plants (**Figure 3**). In recent years, the increasing number of genome sequences coupled with resequencing technology were largely accelerate the investigation of the evolution and phylogenetic relationships of populations in many crops, including rice, soybean, maize, tea plant, etc. (Lai et al., 2010; Jiao et al., 2012; Huang et al., 2012a; Huang et al., 2012b; Huang et al., 2013; Zhou et al., 2015; Varshney et al., 2017; Varshney et al., 2019; Wang et al., 2020). Population genomic analysis using 190 Camellia accessions uncovered independent evolutionary histories between *CSS* and *CSA* (Zhang et al., 2021). Phylogenetic analysis of resequencing 81 diverse accessions of tea plants supported the classification into three differentiated populations, including *CSS*, *CSA*, and wild types (Xia et al., 2020b). This technology was also used to investigate the phylogenetic relationships between cultivated and wild rice accessions (Huang et al., 2012a). However, the high cost of sequencing prevents its further application in more species. Next-generation sequencing (NGS) and new relevant computational tools have allowed for high-throughput sequencing to become more commonplace. As one of the most popular areas in NGS (Strickler et al., 2012), the data are multipurpose and can be used to detect genes (Bräutigam et al., 2010; Chen et al., 2017), single-nucleotide polymorphisms (SNPs) (Scaglione et al., 2012), look at gene expression (Chen et al., 2017), and so on. Our efforts are to construct a transcriptome-based SNP-calling method for intraspecific relationship analysis in *Camellia sinensis*. Our results uncovered that transcriptome sequencing is not only a cost-effective method, but also could provide sufficient genetic information for resolving the intraspecies relationships. We reconstructed robust phylogenetic relationships of *C. sinensis* samples by 99,115 high-quality SNPs from the coding region and uncovered that the sources of tea planted in the Xinyang area were extensive and complex. Here we provided a convenient and accurate method for intraspecies relationship resolution, which will lay a foundation for excavating valuable wild germplasm resources of the tea plant.

### 4.4 Functional investigation of the selection signals reveals the genetic basis behind populations of *CSS* and *CSA*

The tea plant (*Camellia sinensis*) is divided into two varieties, *C. sinensis* var. *sinensis* (*CSS*) and var. *assamica* (*CSA*). They have significant differences in plant height, leaf shape, secondary metabolites, which led to the different processing suitability of tea. China has a wealth of tea germplasms due to its long history of tea plant cultivation and utilization of nearly two thousand years (Xia et al., 2020a). However, the wild ancestor tea plants have yet to be found to date, making the functional analyses of the domestication-related genes still poorly understood. In addition, the influences of ecological and artificial selection make domestication a dynamic and ongoing process (Meyer et al., 2012). In order to find positive selection signals, we expanded the range of significant genes with VCFtools and XP-CLR. Compared with *CSA*, *CSS* is more tolerant to cold stress and can be cultivated over a relatively wide area. Among 53 selected genes, KEGG results showed that some genes were significantly enriched in response to abiotic stress. For example, *CSS0000475* has the activity of acetaldehyde dehydrogenase. In potato studies, it was found that the homologous gene is regulated by methylation to cope with cold stress (Guo et al., 2020). Differences in the processing suitability of the *CSS* and *CSA* were mainly caused by the differentiation of intracellular secondary metabolites. We also confirmed that multiple genes were enriched in metabolic pathways, biosynthesis of secondary metabolites, amino acid metabolism, carbohydrate metabolism, vitamin metabolism, terpenes and polyketones, sugar biosynthesis, and lipid metabolism. GO terms showed significant enrichment in macromolecular modification, endogenous stimulus response and hormonal response, which confirmed the differences between *CSS* and *CSA* in metabolite synthesis and regulation. The differences between *CSS* and *CSA* in photosynthetic intensity and adaptability to the environment might be related to phosphorylation, which may play an important role in photosynthesis, anatomical structure formation and drought resistance (Wang et al., 2013; Liu et al., 2016). For instance, macromolecular modification involves phosphorylation (*CSS0003026*, *CSS0027271*, *CSS0037814*), REDOX (*CSS0031866*), flavonoid synthesis (*CSS0035789*) and DNA methylation (*CSS0045929*). KEGG and GO annotation enrichment analyses were also performed for 31 genes screened by VCFtools and 51 genes screened by XP-CLR, respectively. The differentiation of *CSS* population involves the expression of defense-related genes, which are also significantly enriched in the biosynthesis of metabolites such as arginine, unsaturated fatty acids, α-linolenic acid, and inositol phosphate. *CSA* differentiation was mainly concentrated on genes related to alkaloid and aromatic chemical metabolism and biosynthesis, including glutathione, purine and pyrimidine metabolism. These findings may give clues to explore the domestication traits in *CSS* and *CSA*, especially with the content of galloylated cis-catechins as the most recognized domestication-associated traits in tea plants (Li et al., 2022).

Furthermore, population genetics and transcriptomic analyses revealed that the secondary metabolites and amino acids synthesis-related genes in *CSS* populations were stronger in *CSA* populations but not significant, which was not totally consistent with previous studies (Wang et al., 2020). The reasons for the increased genetic diversity of *CSS* could be as follows: 1) continuous infiltration from ancient local races to self-incompatible cultivated races during the long period of cultivation; 2) adaptation of tea plants to a new environment during propagation, possibly due to strong positive selection or acclimation selection; 3) the tea plant populations were mainly collected in a particular tea growing area. These hypotheses need to be further evaluated. It also confirmed the complexity of genetic diversity in tea plant populations and deserved more attention and research. Extensive sampling of wild and ancient tea trees is necessary to trace the origins of tea trees and determine whether these genes were truly selected during domestication or were merely genetic hitchhikers in other regions of artificial selection.

## 5 Conclusion

Here, we present 94 newly sequenced transcriptomes of *Camellia sinensis*. A total of 99,115 high-quality SNPs were identified in the coding region. We successfully resolved the intraspecies relationship of sampled *C. sinensis* accessions by concatenating the variants. Compared to the phylogeny with low resolution inferred from more than thousands of low-copy nuclear genes, our study showed that the transcriptome-based SNP calling method is effective for untangling intraspecific relationships. The phylogeny and PCA analyses indicate extensive and complex sources of tea plants in the Xinyang area with a long history of tea cultivation. In addition, we speculated the Shihe District and Gushi County as the two earliest tea planting areas in Xinyang. Our study provides an effective method for untangling intraspecific relationships based on transcriptomic data SNP-calling in *Camellia sinensis*, through which we classify the unknown tea plants to *CSS* or *CSA* accurately. Furthermore, we identified some domesticated-related genes involved in regulating secondary metabolite synthesis and trichome formation. These results will provide a significant theoretical basis for processing suitability and will lay a foundation for investigating valuable wild germplasm resources of tea plants.

## Data availability statement

The datasets presented in this study can be found in online repositories. The names of the repository/repositories and accession number(s) can be found in the article/**Supplementary Material**. The newly generated RNA-seq data of 94 *Camellia sinensis* accessions were available in NCBI under accession ID PRJNA850466.

## Author contributions

Conceptualization and supervision: YZ and LC; analysis: ML, YZ, YW, LC, QH, ZQ, and YH; collecting samples: LC, WZ, LQ, and AG; writing original draft preparation: LC and ML; writing review and editing: YZ, LC, ML, ZZ, TL, SSL, LT, DL, HY, SL, and TBS. All authors contributed to the article and approved the submitted version.

## Supplementary material

Supplementary Figure 1 The flowchart for transcriptome-based SNP calling, phylogeny analysis and selection signals identification.

Supplementary Figure 2 The KEGG and GO annotation and enrichment analyses of 31 genes under positive natural selection (top 10% Fst values) were identified based on per-site by VCFtools. **(A)** The KEGG annotation for 31 genes with strong selection signals and the size of the star represents the number of genes, and the node color represents connectivity. **(B)** The X-axis shows gene numbers, and the Y-axis lists the categories in plant GO slim terms. Red, blue and green bars represent biological processes, molecular functions and cellular components, respectively.

Supplementary Figure 3 The KEGG and GO annotation and enrichment analyses of genes located in the strong positive natural selection in genomic blocks by XP-CLR. **(A)** The size of the star represents the number of genes, and the node color represents connectivity. The pathway terms with red color enriched are shared by *Camellia sinensis* var. *assamica* (CSA) and *Camellia sinensis* var. *sinensis* (CSS). The pathway terms with blue and black colors represent CSA-specific and CSS-specific, respectively. **(B)** The X-axis shows gene numbers, and the Y-axis lists the categories in plant GO slim terms. Red, blue and green bars represent biological processes, molecular functions and cellular components, respectively. The GO terms with blue and black color indicate *Camellia sinensis* var. *assamica* specific and *Camellia sinensis* var. *sinensis*, respectively.

Supplementary Figure 4 The analysis of gene family and identification of gene duplication events (GDs) for those 53 genes under positive natural selection were identified both by single locus and genomic blocks. **(A)** Copy number variation of 51 gene families (homologs) across 11 genomes. The gene copy numbers for the CSS and CSA were shown in the purple and blue dotted boxes, respectively. The numbers represent the gene family copy number in the heatmap and the asterisk (*) indicates that the copy number of CSS varies largely compared to those in CSA. *CSS-SCZ*, CSS ‘Shuchazao’; *CSS-LJ43, CSS* ‘Longjing43’; *CSS-BY*, CSS ‘Biyun’; *CSS-HD*, CSS ‘Hangdan’; *CSS-TYG*, CSS ‘Tieguanyin’; *CSA-YK10*, CSA ‘Yunkang 10’; *C. lanceoleosa*, *Camellia lanceoleosa*; *C*. *oleifera, Camellia oleifera* var. *‘Nanyongensis’*; *C. DASZ*, *Camellia* DASZ; *A. chinensis, Actinidia chinensis*; *R. simsii, Rhododendron simsii.* **(B)** The heatmap for the number of gene duplications at different species tree nodes (node ID as phytos). The blue and yellow dotted boxes represent the number of gene duplication events for the genomes of CSS and *CSA*. **(C)** The heatmap represents the number of GDs occurred within the species level. The GD numbers of CSS and CSA were shown in the purple and blue dotted boxes, respectively.
