# Supplementary figures and images for: Transcriptome-based variations effectively untangling the intraspecific relationships and selection signals in Xinyang Maojian tea population
Source: Front Plant Sci. 2023 Feb 20;14:1114284. doi: 10.3389/fpls.2023.1114284 (PMC9986275; doi:10.3389/fpls.2023.1114284)

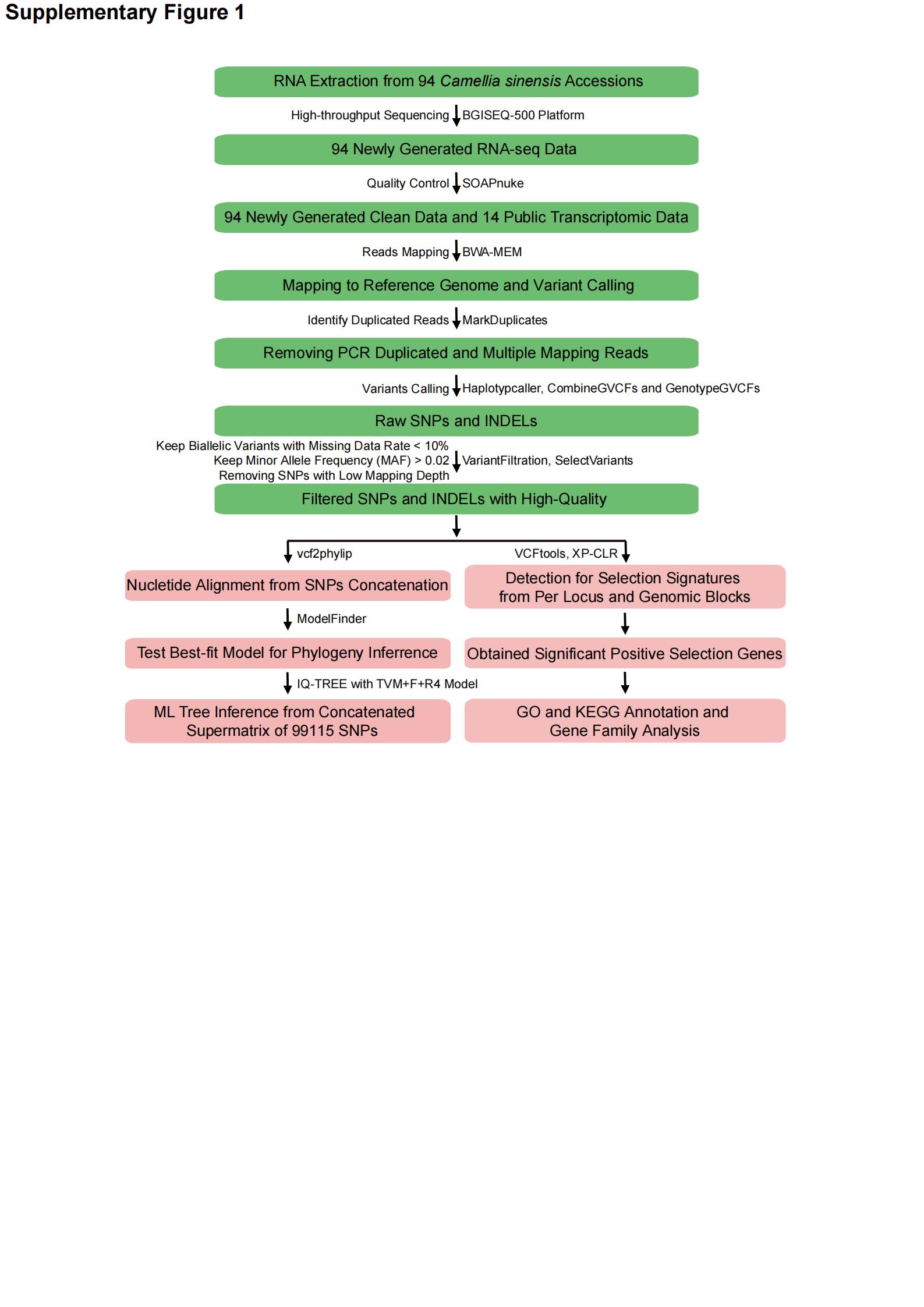

Supplement: Supplementary Figure 1 — The flowchart for transcriptome-based SNP calling, phylogeny analysis and selection signals identification. [file Image_1.jpeg]

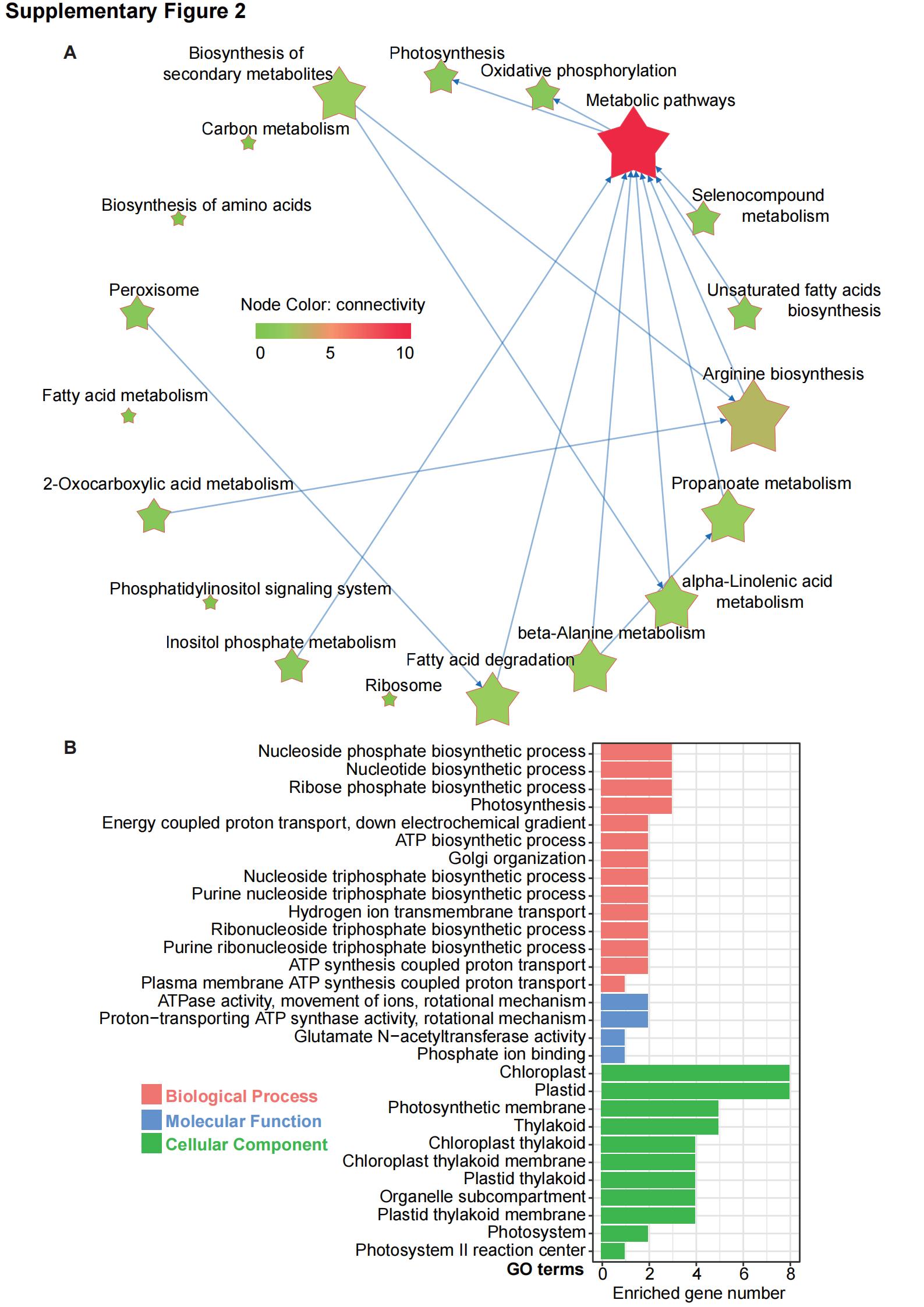

Supplement: Supplementary Figure 2 — The KEGG and GO annotation and enrichment analyses of 31 genes under positive natural selection (top 10% Fst values) were identified based on per-site by VCFtools. (A) The KEGG annotation for 31 genes with strong selection signals and the size of the star represents the number of genes, and the node color represents connectivity. (B) The X-axis shows gene numbers, and the Y-axis lists the categories in plant GO slim terms. Red, blue and green bars represent biological processes, molecular functions and cellular components, respectively. [file Image_2.jpeg]

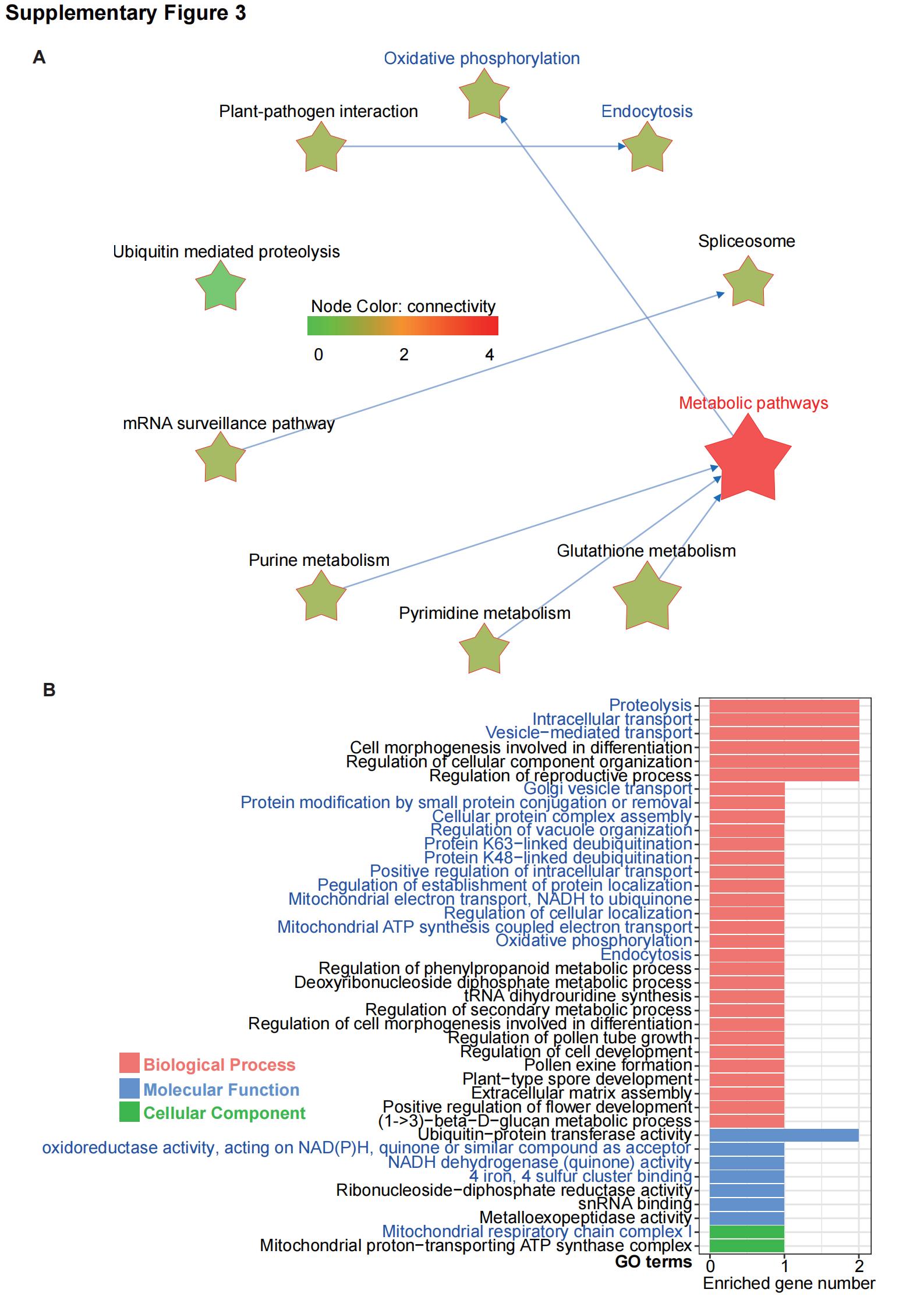

Supplement: Supplementary Figure 3 — The KEGG and GO annotation and enrichment analyses of genes located in the strong positive natural selection in genomic blocks by XP-CLR. (A) The size of the star represents the number of genes, and the node color represents connectivity. The pathway terms with red color enriched are shared by Camellia sinensis var. assamica (CSA) and Camellia sinensis var. sinensis (CSS). The pathway terms with blue and black colors represent CSA-specific and CSS-specific, respectively. (B) The X-axis shows gene numbers, and the Y-axis lists the categories in plant GO slim terms. Red, blue and green bars represent biological processes, molecular functions and cellular components, respectively. The GO terms with blue and black color indicate Camellia sinensis var. assamica specific and Camellia sinensis var. sinensis, respectively. [file Image_3.jpeg]

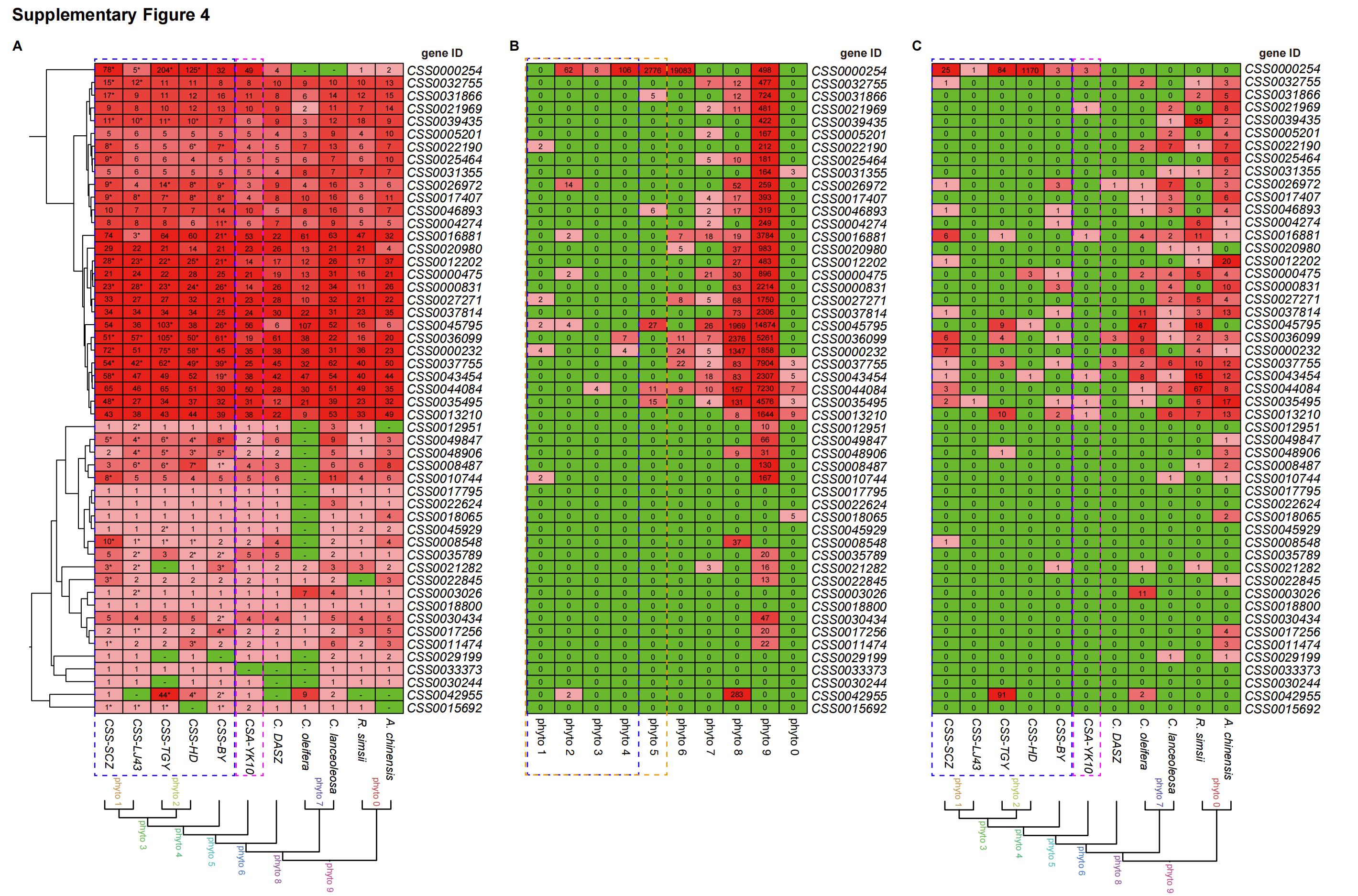

Supplement: Supplementary Figure 4 — The analysis of gene family and identification of gene duplication events (GDs) for those 53 genes under positive natural selection were identified both by single locus and genomic blocks. (A) Copy number variation of 51 gene families (homologs) across 11 genomes. The gene copy numbers for the CSS and CSA were shown in the purple and blue dotted boxes, respectively. The numbers represent the gene family copy number in the heatmap and the asterisk (*) indicates that the copy number of CSS varies largely compared to those in CSA. CSS-SCZ, CSS ‘Shuchazao’; CSS-LJ43, CSS ‘Longjing43’; CSS-BY, CSS ‘Biyun’; CSS-HD, CSS ‘Hangdan’; CSS-TYG, CSS ‘Tieguanyin’; CSA-YK10, CSA ‘Yunkang 10’; C. lanceoleosa, Camellia lanceoleosa; C. oleifera, Camellia oleifera var. ‘Nanyongensis’; C. DASZ, Camellia DASZ; A. chinensis, Actinidia chinensis; R. simsii, Rhododendron simsii. (B) The heatmap for the number of gene duplications at different species tree nodes (node ID as phytos). The blue and yellow dotted boxes represent the number of gene duplication events for the genomes of CSS and CSA. (C) The heatmap represents the number of GDs occurred within the species level. The GD numbers of CSS and CSA were shown in the purple and blue dotted boxes, respectively. [file Image_4.jpeg]
